# Secukinumab treatment in rheumatoid arthritis is associated with incremental benefit in the clinical outcomes and HRQoL improvements that exceed minimally important thresholds

**DOI:** 10.1186/1477-7525-12-31

**Published:** 2014-03-05

**Authors:** Vibeke Strand, Mark Kosinski, Ari Gnanasakthy, Usha Mallya, Shephard Mpofu

**Affiliations:** 1 Stanford University Division of Immunology/Rheumatology, Palo Alto, CA, USA; 2 QualityMetric Incorporated, 24 Albion Road, Lincoln, RI, USA; 3 Novartis Pharmaceuticals Corporation, New Jersey, East Hanover, USA; 4 Novartis Pharma AG, Basel, Switzerland

## Abstract

**Background:**

The primary aim of rheumatoid arthritis (RA) treatment is to induce remission, the absence of disease activity. The objective of this study was to explore the association between clinical endpoints used to gauge RA treatment efficacy and patient-reported outcomes of health-related quality of life, fatigue, and physical function in RA patients treated with secukinumab in a phase 2 randomized controlled trial (RCT).

**Method:**

Adult RA patients (n = 237) with incomplete responses to methotrexate were randomized equally to receive monthly s.c. injections of secukinumab 25 mg, 75 mg, 150 mg, 300 mg or placebo. Clinical endpoints used in this study included the ACR response criteria and its components and simplified disease activity score. Patient-reported outcomes (PRO) included Health Assessment Questionnaire-Disability Index (HAQ-DI), Medical Outcomes Study Short Form-36 [SF-36] Survey, and Functional Assessment of Chronic Illness Therapy-Fatigue (FACIT-Fatigue). Patients were categorized into mutually exclusive groups according to the magnitude and direction of change from baseline to week 16 in each clinical endpoint. Definitions of minimal important differences [MID] in each clinical endpoint were used to categorize patients, as well as thresholds beyond MID. Mean changes from baseline to week 16 were computed for each PRO and analyses of variance to test the differences in PRO changes observed across groups of patients that differed in each clinical endpoint. Analyses were limited to patients randomized to secukinumab treatment. All dose groups were combined (n = 187).

**Results:**

Mean changes from baseline in each PRO differed significantly across groups of patients in the expected direction. With few exceptions, there was considerable agreement between clinical endpoints and PROs concerning the magnitude of change defined as clinically meaningful. More importantly, results demonstrated that greater improvements in clinical endpoints were associated with incrementally better improvements in HRQoL, fatigue, and physical function.

**Conclusion:**

Results of this study show considerable agreement between minimal thresholds of improvement established for PROs and clinical outcome measures used in RA treatment studies and provide thresholds to be considered in gauging the importance of a treatment effect that goes beyond what is considered as minimally important for PRO measures.

## Background

Rheumatoid arthritis (RA) is a systemic, chronic inflammatory disease characterized by joint inflammation and structural damage symmetrically in the hands and feet and large joints. It affects approximately 0.5-1% of the population in developed countries [1-3]. The natural course of the disease is one of persistent symptoms, varying in intensity, with a progressive loss of joint integrity resulting in impairments in physical function. The progression of RA places an enormous burden on the patients, their families, and society as a whole. The annual direct costs of care attributable to RA from the societal perspective was estimated to be $3.6 billion [4] and as the disease progresses, patients often experience an increase in functional impairment that often leads to work disability [5-8]. In addition, patients with RA are at a greater risk of early death [9]. It is estimated that RA reduces the lifespan of patients by anywhere from 3 to 12 years [10].

The disease course of RA varies greatly across individuals. Some individuals experience mild short-term symptoms, but in most cases the disease is progressive for life. The progressive nature of the disease due to high inflammatory disease activity has a profound effect on the individual’s health-related quality of life (HRQoL), including physical functioning, vitality, psychological well-being, and social and emotional roles [11-13]. Accordingly, the goals of RA treatment not only include minimizing the clinical symptoms such as pain and swelling, decreasing disease activity, preventing structural damage, but also the maintenance or improvement in an individual’s functional capacity and health-related quality of life [14]. Because it is known that many of the laboratory measures and clinical markers of disease activity and progression, such as swollen joint counts, C-reactive protein, or erythrocyte sedimentation rates, do not correlate well with patient functional status and are not reliable predictors of long-term outcomes [15,16], it is important to utilize HRQoL and physical function measures to capture the chronic and disabling nature of RA and quantify the long-term impact of the disease and its treatment. Additionally, HRQoL and physical function measures provide useful benchmarks to evaluate the efficacy of RA treatment that are not fully captured by laboratory and clinical markers.

In general, results of randomized controlled trials (RCTs) of RA report clinical endpoints, physical function and HRQoL independently when evaluating treatment efficacy. Given the complementary role of these measures in understanding efficacy of treatment, it is useful to know which benefits in HRQoL and physical function are associated with improvements in well accepted clinical endpoints of RA treatment efficacy. In this study we link mean changes in SF-36 and FACIT-Fatigue to changes in the ACR response criteria and its components [patient and physician global assessment of disease activity and pain intensity ratings] and simplified disease activity index [SDAI] that were used to gauge the efficacy of secukinumab treatment in a phase 2 randomized controlled trial [RCT] of RA patients. Mean changes in HAQ-DI scores were linked to changes in patient and physician assessment of disease activity and SDAI. In addition, we expand on the definition of “response” in each clinical endpoint to evaluate whether incremental improvements beyond what has been established as clinically meaningful results in incremental improvements in HRQoL, physical function, and fatigue.

## Methods

Regulatory and ethical review board approvals from competent authorities in each country were obtained for the study protocol. For a list of independent ethics committees and institutional review boards which approved this study, please see Additional file 1. All patients signed an informed consent document, and the study was conducted in accordance with the Declaration of Helsinki and followed good clinical practice guidelines.

### Study population

237 adults with active RA despite treatment with methotrexate [MTX] for ≥3 months, at doses ≥7.5 mg/week to ≤25 mg/week for at least 4 weeks participated in a 52-week, multi-center RCT to assess the efficacy, safety and tolerability of subcutaneous secukinumab added to MTX. Eligible patients met the ACR 1987 revised RA classification criteria for at least 3 months and were required to have ≥6 of 28 tender joints and ≥6 of 28 swollen joints, hsCRP ≥10 mg/L and/or ESR ≥28 mm/1st hour at randomization. These analyses were conducted on the subset of patients randomized to receive one of four secukinumab doses (n = 187).

### Clinical endpoints

The American College of Rheumatology 20/50/70% Response Criteria was utilized as the primary endpoint of efficacy. In these analyses, ACR-N was used to derive criterion groups for response [17-19]. These analyses also correlated responses by Simplified Disease Activity Index [SDAI]: a simple numerical sum of swollen joint and tender joint counts, patient global assessment of disease activity [PtGA], physician global assessment of disease activity [MDGA], and C-reactive protein [20]. As part of the ACR response criteria and SDAI, the physician global assessment of disease activity (MDGA) and patient global assessment of disease activity (PtGA) were performed using a 100 mm visual analogue scale (VAS) ranging from “very good” to “very poor”, after the questions “Considering all the ways rheumatoid arthritis affects your patient, how would you rate his or her condition today?” (MDGA) and “Considering all the ways that your rheumatoid arthritis affects you, how would you rate your condition today?” (PtGA). Lastly, pain was measured using a 100 mm VAS ranging from “no pain” to “unbearable pain”.

### Physical function

Physical function was measured at baseline and weeks 2, 4, 8, 12 and 16 using the standard version of the Health Assessment Questionnaire Disability Index (HAQ-DI) [21], which queries the difficulty in performing 8 common activities of daily living, scored from 0 (without any difficulty) to 3 (unable to do) with a correction for aids or devices used.

### Health related quality of life

HRQoL was measured at baseline and weeks 2, 4, 8, 12 and 16 using the SF-36v2 Health Survey (SF-36) [22,23]. It includes 36 questions that are aggregated to score eight domains: physical functioning (PF), role limitations due to physical health (RP), bodily pain (BP), general health perceptions (GH), vitality (VT), social functioning (SF), role limitations due to emotional health (RE), and mental health (MH). Physical (PCS) and mental (MCS) component summary scores are derived from the eight SF-36 domains, with normative scores of 50 and standard deviations of 10.

### Fatigue

Fatigue was measured at baseline and weeks 2, 4, 8, 12 and 16 using the Functional Assessment of Chronic Illness Therapy-Fatigue (FACIT-Fatigue). The FACIT-Fatigue consists of 13 items that assesses self-reported fatigue and its impact upon daily activities and function, over the past 7 days; each item is scored on a 5-point Likert scale (0 to 4) [24-26].

### Statistical analyses

A known-groups validity [27] approach was taken to explore the association between changes in HAQ-DI, SF-36, and FACIT-Fatigue scores and changes in each of the clinical endpoints. Changes in clinical endpoints and HAQ-DI, SF-36, and FACIT-Fatigue scores were derived by subtracting baseline values from week 16 values. Using the known-groups validity [27] approach mean changes in HAQ-DI, SF-36, and FACIT-Fatigue scores were compared across mutually exclusive groups of patients “responding” according to the following clinical endpoints:

– ACR Improvement (ACR-N).

– Simplified Disease Activity Index (SDAI).

– Patient reported pain.

– Patient global assessment of disease activity.

– Physician global assessment of disease activity.

Four mutually exclusive categories of patients were derived from ACR-N at week 16. Group 1 consisted of patients whose ACR-N was <20 [“non-responders”]; Group 2: ACR-N 20 – 49; Group 3: ACR-N 50–69 and group 4: ACR-N ≥70. Analyses of variance tested the statistical significance of differences in mean changes in SF-36 and FACIT-Fatigue scale scores across these four ACR-N patient groups.

Using criteria developed for interpreting changes in SDAI patients were categorized into four mutually exclusive groups [28]. The development and validation of SDAI cutoff scores are well documented elsewhere [20,29]. Group 1 consisted of patients whose change in SDAI score was > 10 points (worsening); Group 2: within + and – 10 points (same); Group 3: -10 to -21 points (minor improvement) and group 4: -22 points or more (major improvement). Analyses of variance tested the statistical significance of differences in mean changes in SF-36, FACIT-Fatigue, and HAQ-DI scale scores across these four SDAI patient groups.

Patients were additionally grouped into five mutually exclusive categories based on the magnitude and direction of change in scores from baseline to week 16 in PtGA and VAS pain. Improvements of ≥10 points in both PtGA and VAS pain measures have been defined as clinically meaningful [30-32]. Using these criteria, patients were categorized in the following manner. Group 1 consisted of patients whose change in score >10 points (worse); Group 2: within +10 and -10 points (same); Group 3: -10 to -20 points (“minimal” improvement); Group 4: -21 to -40 points (“moderate” improvement) and group 5: > - 40 points (“major” improvement). Analyses of variance tested the statistical significance of changes in mean SF-36, FACIT-Fatigue, and HAQ-Di scores across these five patient groups derived from these criterion measures.

A similar change in MDGA was considered clinically meaningful. Patients were grouped into five categories as above, and analyses of variance tested the statistical significance of changes in mean SF-36, FACIT-Fatigue, and HAQ-DI scores across these five patient groups derived from the MDGA.

## Results

Table 1 presents baseline demographics, clinical characteristics, and HRQoL, physical function and fatigue scores for patients who were randomized to one of four secukinumab dose groups. The majority of the sample was female (79.7%) and Caucasian (73.3%). The average age was 54.9 years. Mean baseline scores on VAS pain (53.6), PtGA (61.6) and HAQ-DI (1.49) as well as MDGA (61.9), SJC (11.4) and TJC (14.7) were all indicative of active disease. Similarly, mean baseline HRQoL scores were indicative of significant disease impact. For example, SF-36 domain and summary scores were much lower than normative values observed in the general population: PCS approximately 2 SDs less, and MCS scores 1 SD lower [33].

**Table 1:** **Demographics**, **clinical characteristics**, **and baseline HRQoL scores of participants randomized to four secukinumab dose groups** (**N** = **187**)

**Demographics**	
Female (n, %)	149 (79.7)
Age (mean, SD)	54.9 (10.8)
Caucasian (n, %)	137 (73.3)
Clinical assessment	
Prior use of biologics (n, %)	38 (20.3)
VAS pain intensity (mean, SD)	53.6 (20.8)
SDAI (mean, SD)	40.3 (11.9)
Patient global assessment disease activity (mean, SD)	61.6 (19.1)
Physician global assessment disease activity (mean, SD)	61.9 (14.9)
Swollen joint count (mean, SD)	11.4 (4.8)
Tender joint count (mean, SD)	14.7 (6.2)
HRQoL scales	
SF-36 Physical functioning (mean, SD)	40.5 (21.7)
SF-36 Role physical (mean, SD)	42.8 (24.3)
SF-36 Bodily pain (mean, SD)	36.1 (16.9)
SF-36 General health (mean, SD)	40.4 (16.6)
SF-36 Vitality (mean, SD)	39.0 (19.7)
SF-36 Social functioning (mean, SD)	57.2 (26.1)
SF-36 Role emotional (mean, SD)	54.1 (26.7)
SF-36 Mental health (mean, SD)	56.8 (19.4)
SF-36 Physical summary (mean, SD)	33.8 (7.5)
SF-36 Mental summary (mean, SD)	40.5 (11.0)
HAQ-DI (mean, SD)	1.49 (0.6)
FACIT-Fatigue (mean, SD)	28.3 (10.1)

Table 2 presents mean changes in HRQoL and fatigue scores across groups of patients that differed in ACR-N. As shown, mean changes in HRQoL and fatigue scores differed significantly across the four ACR-N groups in the expected direction. Patients in Group 4 (ACR-N ≥ 70) reported the largest improvements in scores across all HRQoL domains and fatigue, followed by Group 3 (ACR-N 50–69), and then Group 2 (ACR-N 20–49), with little or no change in HRQoL and fatigue scores in Group 1. SF-36 domains of BP, VT, GH and PCS summary score and FACIT showed the greatest differences in mean score changes across the four ACR-N groups as indicated by the magnitude of F-statistics.

**Table 2:** **Mean changes in HRQoL scores from baseline to study phase completion** (**week 16) by ACR improvement categories, all dose groups combined**

	**ACR < 20% (n = 100)**	**ACR 20-49% (n = 49)**	**ACR 50-69% (n = 24)**	**ACR 70% (n = 9)**	**F**	**p-value**
**PRO Instruments**						
SF-36						
Physical functioning	2.1 (15.6)	8.5 (17.2)	17.9 (18.5)	24.4 (28.4)	9.1	0.0000
Role physical	2.7 (17.3)	12.8 (22.5)	23.9 (23.5)	27.8 (35.2)	10.2	0.0000
Bodily pain	0.4 (14.7)	18.1 (16.6)	24.0 (19.0)	39.1 (13.2)	33.1	0.0000
General health	-1.9 (12.7)	4.7 (12.8)	15.9 (13.6)	18.7 (11.3)	17.7	0.0000
Vitality	0.1 (16.1)	9.9 (19.6)	18.5 (21.1)	29.9 (24.8)	13.2	0.0000
Social functioning	-1.5 (23.9)	12.8 (21.9)	7.8 (27.0)	29.2 (37.0)	7.0	0.0000
Role emotional	-2.1 (22.5)	8.9 (27.2)	13.5 (26.6)	29.6 (27.0)	7.1	0.0000
Mental health	-0.2 (15.2)	6.8 (16.5)	9.4 (23.6)	23.9 (23.0)	7.2	0.0000
Physical summary	0.8 (4.9)	4.6 (5.9)	9.3 (6.1)	10.1 (10.3)	20.8	0.0000
Mental summary	-1.0 (8.9)	4.0 (9.3)	4.3 (12.9)	13.7 (16.0)	8.1	0.0000
FACIT-Fatigue	0.6 (7.2)	5.7 (7.2)	8.8 (8.0)	11.3 (4.5)	14.9	0.0000

Mean changes in HRQoL, fatigue and physical function scores differed significantly across the groups of patients who differed in magnitude of changes in SDAI in the expected direction (Table 3). Patients grouped according to an increase in disease activity (SDAI change > +10 points) showed relatively large decreases in SF-36 and FACIT scores (worsening) as well as increases in HAQ-DI score (worsening). Patients categorized as having little or no meaningful change in disease activity (SDAI change between +/-10 points) reported relatively small or no changes in HRQoL, fatigue and physical function. Patients categorized as having “minor” (SDAI changes from -10 to -21 points) and “major” (SDAI changes -22 points or more) improvements in disease activity reported clinically meaningful improvement in HRQoL, fatigue and physical function. As shown in Table 3, there were incremental improvements in SF-36, FACIT, and HAQ-DI scores going from “minor” improvement to “major” improvements in SDAI. With few exceptions, reported improvements in SF-36, FACIT, and HAQ-DI scores nearly doubled for patients in the “major” compared to the “minor” improvement group. SF-36 domains of BP, PF, RP, VT, GH, SF and RE as well as PCS scores showed the greatest differences in mean change scores across the four SDAI groups as indicated by the magnitude of the F-statistics. Similarly, FACIT and HAQ-DI showed very large differences in mean change scores across the four SDAI groups.

**Table 3:** **Mean changes in HRQoL scores from baseline to study phase completion** (**week 16) by categories of change in SDAI, all dose groups combined**

	**SDAI Categories of change**					
	**> +10 (n = 4)**	**+10 to -10 (n = 67)**	**-10 to -21 (n = 57)**	**-22 to -57 (n = 53)**	**F**	**p-value**
**PRO Instruments**						
SF-36						
Physical functioning	-7.5 (40.9)	2.5 (14.3)	6.5 (16.5)	14.8 (20.2)	5.8	0.0008
Role physical	0.0 (15.3)	1.7 (18.9)	9.5 (20.3)	20.5 (24.2)	8.2	0.0000
Bodily pain	-13.0 (11.2)	0.4 (14.5)	11.4 (17.0)	23.3 (19.9)	20.6	0.0000
General health	-10.0 (17.3)	-1.1 (13.5)	3.1 (13.7)	10.2 (13.8)	8.0	0.0001
Vitality	-1.6 (25.2)	0.2 (16.8)	5.0 (18.3)	17.5 (20.9)	8.9	0.0000
Social functioning	-28.1 (21.3)	-0.4 (21.4)	3.7 (26.0)	16.3 (26.7)	7.3	0.0001
Role emotional	-14.6 (14.2)	-0.4 (22.6)	5.1 (26.6)	11.6 (28.2)	3.0	0.0332
Mental health	-1.3 (7.5)	-1.0 (15.2)	5.4 (18.5)	9.4 (20.3)	3.6	0.0156
Physical summary	-3.0 (10.5)	0.8 (4.6)	3.2 (5.4)	7.6 (7.1)	15.5	0.0000
Mental summary	-4.7 (9.6)	-0.7 (8.5)	1.9 (10.6)	5.3 (12.1)	3.8	0.0108
HAQ-DI	0.16 (0.07)	-0.04 (0.31)	-0.25 (0.39)	-0.53 (0.52)	15.6	0.0000
FACIT-Fatigue	-6.8 (5.2)	0.6 (7.2)	3.5 (7.3)	8.3 (7.3)	14.1	0.0000

Table 4 presents mean changes in SF-36, FACIT, and HAQ-DI scores across categories of patients that differed in the magnitude of change in VAS pain scores, which differed significantly in the hypothesized direction across the five categories of change. On average, patients categorized in the group that reported increases in pain showed a general worsening in SF-36, FACIT, and HAQ-DI scores. Mean improvements in SF-36, FACIT, and HAQ-DI increased incrementally with greater reported reductions in pain. SF-36 domains measuring BP, PF, RP, and VT showed the greatest differences in mean score changes across the categories of changes in pain, as did FACIT and HAQ-DI.

**Table 4:** **Mean changes in HRQoL scores from baseline to study phase completion** (**week 16**) **by categories of change in pain ratings**, **all dose groups combined**

	**Categories of change in pain ratings**						
	**> +10 (n = 31)**	**+10 to -10 (n = 66)**	**-11 to -20 (n = 37)**	**-21 to -40 (n = 28)**	**< -40 (n = 20)**	**F**	**p-value**
**PRO Instruments**							
SF-36							
Physical functioning	-2.0 (19.6)	1.7 (13.7)	8.9 (19.7)	13.2 (14.4)	20.3 (22.9)	6.1	0.0001
Role physical	-1.5 (19.7)	4.5 (18.1)	11.0 (20.1)	19.2 (20.6)	26.3 (30.1)	8.0	0.0000
Bodily pain	-7.3 (13.9)	4.0 (13.4)	15.1 (15.0)	20.5 (16.3)	33.9 (22.3)	28.4	0.0000
General health	-4.4 (11.0)	0.6 (13.8)	3.6 (12.8)	7.1 (14.6)	17.8 (13.1)	9.8	0.0000
Vitality	0.4 (13.9)	-0.3 (15.6)	7.6 (19.4)	12.7 (16.4)	28.8 (27.5)	11.8	0.0000
Social functioning	-6.0 (24.8)	-0.6 (18.9)	9.8 (17.9)	10.7 (28.6)	24.4 (39.4)	6.4	0.0001
Role emotional	-7.8 (18.7)	1.4 (23.6)	5.4 (21.4)	10.0 (29.8)	24.6 (32.2)	5.9	0.0002
Mental health	-2.7 (14.6)	2.4 (15.4)	3.5 (17.4)	8.6 (17.2)	15.5 (27.1)	3.9	0.0047
Physical summary	-0.3 (6.3)	1.1 (4.8)	4.6 (5.7)	6.6 (5.0)	9.9 (1.8)	15.1	0.0000
Mental summary	-2.7 (8.3)	0.3 (8.4)	2.0 (8.3)	3.9 (10.9)	10.4 (17.1)	5.9	0.0002
HAQ-DI	0.08 (0.36)	-0.06 (0.33)	-0.26 (0.36)	-0.46 (0.39)	-0.86 (0.51)	21.9	0.0000
FACIT-Fatigue	0.2 (5.7)	0.6 (7.4)	4.2 (5.0)	8.8 (8.2)	10.7 (9.1)	14.0	0.0000

Table 5 presents mean changes in SF-36, FACIT, and HAQ-DI scores across categories of patients differing in the magnitude of changes in PtGA scores. Significant differences in mean changes in scale scores were observed across the 5 categories of PtGA change, with few exceptions. In general, increased disease activity (increase in PtGA ≥ +10 points) was associated with mean score decreases in SF-36, FACIT, and HAQ-DI. In each of the categories of improvement in PtGA, meaningful changes in SF-36, FACIT, and HAQ-DI were observed. In most instances, the mean score changes in SF-36, FACIT, and HAQ-DI improved incrementally with each incremental improvement in PtGA. SF-36 domains of BP, PF, RP, and VT, as well as FACIT and HAQ-DI showed the greatest differences in mean score changes across the categories of changes in PtGA.

**Table 5:** **Mean changes in HRQoL scores from baseline to study phase completion** (**week 16**) **by categories of change in the patient global assessment of disease activity**, **all dose groups combined**

	**Categories of change in patient global assessment of disease activity**						
	**> +10 (n = 27)**	**+10 to -10 (n = 64)**	**-11 to -20 (n = 27)**	**-21 to -40 (n = 41)**	**< -40 (n = 23)**	**F**	**p-value**
**PRO Instruments**							
SF-36							
Physical functioning	-0.9 (19.7)	3.2 (14.2)	10.9 (19.1)	8.3 (17.3)	18.0 (22.4)	4.2	0.0029
Role physical	-0.6 (14.8)	9.4 (19.4)	9.3 (24.1)	6.9 (19.8)	26.1 (30.5)	5.2	0.0006
Bodily pain	-6.9 (11.4)	4.5 (17.1)	11.7 (17.5)	18.1 (13.6)	29.9 (21.9)	19.9	0.0000
General health	-3.9 (11.9)	0.5 (12.5)	4.4 (16.9)	5.3 (12.2)	14.4 (16.5)	6.7	0.0001
Vitality	-3.9 (13.1)	4.4 (17.7)	8.8 (21.2)	7.2 (18.0)	21.7 (25.7)	6.1	0.0001
Social functioning	-6.9 (19.4)	2.1 (21.1)	5.6 (34.1)	10.7 (18.9)	16.8 (35.7)	3.6	0.0081
Role emotional	-7.1 (16.3)	2.9 (27.3)	7.1 (31.2)	4.9 (20.0)	18.8 (27.9)	3.4	0.0101
Mental health	-1.1 (12.4)	3.0 (17.4)	1.4 (22.3)	6.7 (16.1)	11.9 (21.5)	2.1	0.0827
Physical summary	-0.6 (5.9)	1.9 (4.7)	4.5 (6.0)	4.2 (5.7)	9.4 (8.8)	10.4	0.0000
Mental summary	-2.7 (6.9)	1.2 (10.2)	1.4 (12.9)	2.9 (8.7)	7.1 (13.3)	2.9	0.0219
HAQ-DI	0.01 (0.31)	-0.12 (0.37)	-0.32 (0.4)	-0.35 (0.40)	-0.64 (0.58)	10.4	0.0000
FACIT-Fatigue	-1.1 (6.4)	1.94 (7.5)	5.9 (7.4)	3.78 (6.3)	10.72 (9.1)	9.8	0.0000

Mean changes in HRQoL, physical function and fatigue also differed significantly across the categories of change in MDGA (Table 6). With few exceptions, patients categorized by physicians as having an increase in disease activity (change of +10 points) reported worsening in SF-36 and FACIT scores on average and a slight increase in HAQ-DI scores (worsening). Meaningful improvements in SF-36, FACIT and HAQ-DI scores emerged in the category of improvement representing MDGA changes of -21 to -40 points and increased incrementally with the next category of improvement representing the largest improvement (< -40 points). SF-36 domains of BP, PF and VT, as well as FACIT and HAQ-DI, showed the greatest differences in mean change scores across the categories of change in MDGA.

**Table 6:** **Change in physician global assessment of disease activity**, **all dose groups combined**

	**Categories of change in physician global assessment of disease activity**						
	**> +10 (n = 8)**	**+10 to -10 (n = 44)**	**-11 to -20 (n = 33)**	**-21 to -40 (n = 56)**	**< -40 (n = 43)**	**F**	**p-value**
**PRO Instruments**							
SF-36							
Physical functioning	-5.6 (27.9)	1.6 (13.2)	2.7 (12.6)	10.1 (19.5)	14.3 (19.7)	4.8	0.0010
Role physical	-8.9 (13.0)	1.8 (17.7)	6.3 (19.4)	11.2 (21.2)	21.0 (25.9)	6.4	0.0001
Bodily pain	-6.6 (13.4)	-0.8 (15.7)	6.4 (18.8)	14.3 (14.2)	22.2 (22.3)	12.3	0.0000
General health	-5.9 (17.2)	-1.2 (14.7)	4.2 (12.4)	3.6 (14.6)	8.5 (13.3)	3.4	0.0097
Vitality	0.0 (13.8)	-3.1 (18.5)	1.9 (19.7)	7.4 (13.8)	20.7 (22.1)	10.3	0.0000
Social functioning	-15.6 (23.8)	-3.1 (24.1)	0.0 (22.9)	8.7 (19.9)	16.7 (30.9)	5.7	0.0003
Role emotional	-10.4 (25.5)	-3.4 (23.7)	1.9 (22.9)	7.7 (24.6)	13.3 (28.9)	3.4	0.0109
Mental health	-8.1 (13.1)	-4.0 (15.4)	5.0 (16.2)	4.0 (16.7)	14.5 (19.7)	7.6	0.0000
Physical summary	-1.8 (8.0)	1.0 (4.9)	1.9 (4.9)	4.5 (5.9)	6.5 (7.8)	6.7	0.0000
Mental summary	-4.8 (10.5)	-.27 (9.2)	1.1 (8.9)	2.4 (8.6)	7.5 (12.8)	6.5	0.0001
HAQ-DI	0.03 (0.44)	0.05 (0.33)	-0.14 (0.36)	-0.28 (0.41)	-0.54 (0.53)	9.1	0.0000
FACIT-Fatigue	-4.7 (5.5)	-0.6 (7.8)	2.5 (7.1)	4.1 (6.2)	9.6 (7.2)	14.8	0.0000

## Discussion

For the past 15 years, PRO measures of physical function, HRQoL and fatigue have played an increasingly prominent role in evaluating the safety and efficacy of RA treatment. It is well recognized that these measures provide important complimentary information in understanding the efficacy of treatment beyond traditional clinical endpoints of ACR, DAS and SDAI responses. While most evaluations of the efficacy of recently approved RA treatments present clinical outcomes, additionally citing improvements in physical function, HRQoL and fatigue [34,35], few have tried to link the two types of outcomes together to further our understanding of the patient benefits associated with a response by standard clinical endpoints for RA. The objective of this study was to link changes in several PROs to improvements in ACR and SDAI responses. In addition, analyses were designed to determine whether further incremental benefits in PROs including physical function, HRQoL and fatigue accrue with larger improvements in these clinical endpoints beyond what would be expected as MIDs.

Analyses conducted here went beyond standard correlation analyses to investigate the magnitude of mean changes in physical function, HRQoL and fatigue scores associated with various ranges of change in standard efficacy endpoints in RA RCTs. As expected, mean improvements in HAQ-DI, SF-36 and FACIT scores differed significantly across groups of patients categorized according to their magnitude of change in each clinical endpoint investigated. Very good agreement was observed between what was defined as “clinically meaningful” in each of the clinical endpoints and what is defined as the MID for scales of the SF-36, HAQ-DI, and FACIT instruments. For example, with few exceptions mean changes from baseline in scores across all SF-36 domains and summary scores and FACIT-Fatigue scales among patients in the minimal ACR-N response category (20-49%) met or exceeded MID for these PRO instruments [36,37]. Likewise, mean changes from baseline in HAQ-DI, SF-36 and FACIT met MID among patients with “minor” improvements in SDAI, who were also included in the category representing the smallest meaningful change (-11 to -20) in VAS pain and PtGA. These results mutually validate the cut points established as clinically meaningful for clinical and MID for PROs and highlight that even the smallest benefit observed with treatment in each clinical endpoint is associated with clinically meaningful improvements in physical function, HRQoL and fatigue.

Another key finding from these analyses was there was considerable incremental improvement across all physical function, HRQoL and fatigue scores associated with greater levels of improvement in each clinical endpoint beyond what would be considered of minimal clinical significance. For example, analyses involving ACR-N showed that, as patients met higher thresholds of improvement on ACR-N there were incremental improvements in HRQoL and fatigue. At the first level of meaningful responses by ACR-N (ACR-N category of 20 to 49%) mean changes from baseline in all SF-36 domains and FACIT met established definitions for MID. With few exceptions, the magnitude of mean changes in SF-36 and FACIT doubled and in some instances tripled at the next level of ACR-N responses (ACR-N category 50 to 69) and were of moderate to large effect sizes [38]. Changes of this magnitude could potentially be considered as really important differences (RID) [39]. Lastly, at the highest ACR-N response category (ACR-N ≥ 70) mean changes from baseline in each SF-36 and FACIT scores increased further compared to lower thresholds of ACR-N responses and were all in the range of large effect sizes [38].

A similar pattern of results was observed with analyses based on SDAI, where HAQ-DI could also be included as it is not a component of the SDAI. Mean changes in physical function, HRQoL, and fatigue scores increased incrementally from no meaningful change to “minimal” and from “minimal” to “major” improvements by SDAI. In the “minimal” improvement SDAI category mean changes in HAQ-DI, SF-36 and FACIT scores exceeded MID, with a few exceptions, and the magnitude of changes were in the range of small to moderate effect sizes. Going from “minimal” to “moderate” improvement categories of SDAI, changes from baseline in HAQ-DI, SF-36 and FACIT more than doubled in magnitude and in the “major” improvement category of SDAI were in the range of large effect sizes also considered as ≥ RID.

As expected, significant improvements in physical function, HRQoL, and fatigue were observed with reductions in VAS pain scores. With exception of SF-36 MH domain, mean improvements from baseline exceeded MID even in subjects reporting the smallest category of pain reduction (VAS change of -11 to -20 points). Mean changes from baseline in HAQ-DI, SF-36 and FACIT were generally in the small to moderate effect size range at this category of pain reduction. The magnitude of improvements increased incrementally at the next highest category of pain reduction (VAS change of -21 to -40 points) where they were generally in the moderate effect size range. Finally largest improvements in physical function, HRQOL and fatigue scores were observed at the highest category of pain reduction (VAS change of < -40 points), and were, with few exceptions, in the large effect size range.

Mean changes in physical function, HRQoL and fatigue scores differed significantly across the groups of patients that differed in their level of change defined by PtGA and MDGA. In general, patients with the greatest improvements in PtGA and MDGA also reported the greatest improvements in HRQoL, physical function, and fatigue. However, differences in mean changes from baseline were not always ordered consistently across categories of improvement defined by PtGA. For example, in many instances mean improvements from baseline were either the same or reversed in order of magnitude between the two intermediate categories of improvement (-11 to-20 and -21 to-40) indicating there were few discernible benefits in physical function, HRQoL and fatigue between the two. Comparing the results observed between PtGA and MDGA it appeared that larger improvements in MDGA were required before meaningful changes in physical function, HRQOL and fatigue were reported. With few exceptions, mean changes in physical function, HRQoL and fatigue scores were smaller than the MID threshold on each scale at the first category of improvement (-11 to -20 points) on the physician global assessment, while most changes in physical function, HRQoL and fatigue scores met the MID at the first category of improvement on the patient global assessment. While it has been established that a 10 point improvement in MDGA is clinically meaningful the data from this study suggest that there were no discernible benefits in physical function, HRQoL and fatigue until a change of at least -21 points.

The results of this study may be of importance to those investigators wishing to understand the importance of change in PROs in treatment studies of RA. Specifically, the value that the results of this study lends to investigators using PROs in treatment studies relates to the magnitude of change in PROs that one might expect to observe as treatment results in a greater response on clinical outcomes. With newer treatments developed for RA it has become more commonplace in RA treatment studies to go beyond the minimal threshold of improvement (ACR20 response criteria) to include evaluations of treatment efficacy in terms of ACR50 and ACR70 response thresholds. The results of this study provide potentially useful thresholds of improvement in PROs that go beyond the threshold of minimal importance established for these tools.

A limitation of this study concerns the statistical tests used to assess the statistical significance of differences in mean PRO score changes across groups of patients differing in the magnitude of clinical outcomes. Specifically, in several instances samples sizes were relatively small resulting in large differences in score variances observed across groups, which is a violation of an assumption underlying ANOVA. To address this limitation the analyses of known groups differences were conducted in two alternative ways to assess the robustness of the results determined with ANOVA. First, a non-parametric test, the Kruskal-Wallis test, was conducted. This test makes no assumptions of the equality of variances observed across comparison groups. The results of these analyses were all statistically significant confirming the results of the ANOVA tests. Second, groups with small sample sizes were collapsed with an adjacent category and ANOVA tests were conducted. Results of these analyses were all statistically significant, too. Since the main objective of this study was to determine the magnitude of mean PRO score changes associated with incremental improvement in clinical outcome measures, the original analyses were presented despite the violation of the assumption underlying ANOVA.

## Conclusion

In conclusion, the results of this study demonstrated considerable agreement between changes in clinical endpoints used to evaluate efficacy in RA RCTs and PROs of physical function, HRQoL and fatigue. Using a known-groups validation approach [27] to study the relationship between these two types of outcome measures, these analyses demonstrated that there was considerable agreement in the thresholds established as clinically meaningful changes in both types of measures. More importantly, the results demonstrated that going beyond a threshold of minimal improvement in a clinical endpoint was associated with incremental improvements in HRQoL, physical function and fatigue beyond what would be considered to represent MID.

## Abbreviations

ACR: American College of Rheumatology; BP SF-36: Bodily pain scale; CRP: C-reactive protein; ES: Effect size; FACIT: Functional assessment of chronic illness therapy; GH SF-36: General health scale; HAQ-DI: Health Assessment Questionnaire-Disability Index; HRQoL: Health related quality of life; MCS: Mental component summary scale; MDGA: Physician global assessment of disease activity; MH SF-36: Mental health scale; MID: Minimal important difference; MTX: Methotrexate; PCS SF-36: Physical component summary scale; PF SF-36: Physical functioning scale; PRO: Patient reported outcome; PtGA: Patient global assessment of disease activity; RA: Rheumatoid arthritis; RCT: Randomized control trial; RE SF-36: Role emotional scale; RID: Really important difference; RP SF-36: Role physical scale; SD: Standard deviation; SDAI: Simplified disease activity index; SF SF-36: Social functioning scale; SF-36: Medical Outcomes Study Short-Form 36; TJC: Tender joint count; VAS: Visual analogue scale; VT SF-36: Vitality scale.

## Competing interests

Mr. Gnanasakthy, Dr. Mallya, and Dr. Mpofu are employees of Novartis and own shares of stock from the company. Dr. Strand and Mr. Kosinski have no financial interests related to the work presented in this paper.

## Authors’ contributions

All authors were involved in the drafting of the article or reviewing it critically for important intellectual content. In addition, all authors approved the final draft to be published. Mr. Kosinski had full access to all of the data in the study and takes responsibility for the integrity of the data and the accuracy of the data analysis.

## Supplementary Material

Additional file 1 Appendix 16.1.3 - List of Independent Ethics Committees or Institutional Review Boards. Click here for file
