# Supplementary material for: Secukinumab treatment in rheumatoid arthritis is associated with incremental benefit in the clinical outcomes and HRQoL improvements that exceed minimally important thresholds
Source: Health Qual Life Outcomes. 2014 Mar 5;12:31. doi: 10.1186/1477-7525-12-31 (PMC4016279; doi:10.1186/1477-7525-12-31)
Supplement: Additional file 1 — Appendix 16.1.3 - List of Independent Ethics Committees or Institutional Review Boards. [file 1477-7525-12-31-S1.pdf]

Appendix 16.1.3 - List of Independent Ethics Committees or Institutional Review Boards

Center No.	Ethics Committee or Institutional Review Board	Department / Organization	Address Country
0001	UCL St. Luc	Avenue Hippocrate 55-14, Tour Harvey, niveau 0	Bruxelles 1200 Belgium
0010	EK Revmatologický ústav	Na Slupi 4	Praha 128 50 Czech Republic
0011	Etická komise pro multicentrická hodnocení FN Motol	Fakultní nemocnice v Motole, V úvalu 84	Praha 150 06 Czech Republic
0012	Etická komise pro multicentrická hodnocení FN Motol	Fakultní nemocnice v Motole, V úvalu 84	Praha 150 06 Czech Republic
0013	Etická komise pro multicentrická hodnocení FN Motol	Fakultní nemocnice v Motole, V úvalu 84	Praha 150 06 Czech Republic
0040	Ethik-Kommission der Medizinischen Fakultät der Technischen Universität München Klinikum rechts der Isar	Ismaninger Str. 22	Muenchen 81675 Germany
0041	Ethik-Kommission der Medizinischen Fakultät der Technischen Universität München Klinikum rechts der Isar	Ismaninger Str. 22	Muenchen 81675 Germany
0042	Ethik-Kommission der Medizinischen Fakultät der Technischen Universität München Klinikum rechts der Isar	Ismaninger Str. 22	Muenchen 81675 Germany
0045	Ethik-Kommission der Medizinischen Fakultät der Technischen Universität München Klinikum rechts der Isar	Ismaninger Str. 22	Muenchen 81675 Germany
0047	Ethik-Kommission der Medizinischen Fakultät der Technischen Universität München Klinikum rechts der Isar	Ismaninger Str. 22	Muenchen 81675 Germany
0048	Ethik-Kommission der Medizinischen Fakultät der Technischen Universität München Klinikum rechts der Isar	Ismaninger Str. 22	Muenchen 81675 Germany

Center No.	Ethics Committee or Institutional Review Board	Department / Organization	Address Country
0062	Debreceni Egyetem Orvos- és Egészségtudományi Centrum (DEOEC RKEB/IKEB) Tudományos Bizottságának Regionális és Intézményi Kutatásetikai Bizottsága	Nagyerdei krt. 98.	Debrecen 4032 Hungary
0064	BMKT Pándy Kálmán Kórház Intézeti Kutatásetikai Bizottság	Semmelweis u. 1.	Gyula 5700 Hungary
0065	MÁV Kórház és Rendelőintézet Intézeti Kutatásetikai Bizottság	Verseghy u. 6-8.	Szolnok 5000 Hungary
0080	NHO Sagamihara National Hospital Institutional Reveiw Board	18-1,Sakuradai, Minami-ku	Sagamihara City Kanagawa 252-0392 Japan
0081	Jinbo Orthopedic Hospital Institutional Reveiw Board	5-38-41,Honcho	Koganei city Tokyo 184-0004 Japan
0082	Nakayama Internal Medicine, Rheumatology & Allergy Institutional Reveiw Board	1-7-4,Higashikawasakicho,Chuo-ku	Kobe city Hyogo 650-0044 Japan
0084	Aso Co., Ltd, Iizuka Hospital Institutional Reveiw Board	3-83,Yoshiocho	Iizuka city Fukuoka 820-8505 Japan
0086	Shinagawa East One Medical Clinic Institutional Reveiw Board	2-16-1, Konan	Shinagawa-ku Tokyo 108-0075 Japan
0100	Institutional Review Board / Seoul St. Mary Hospital, The Catholic University of Korea	#505, Banpo-Dong, Seocho-Gu	Seoul 137-040 Korea
0101	IRB / Pusan National University Hospital	Clinical Research Center of Medical Research Institute, Pusan National University Hospital 3Fl., Office Building, 305 Gudeok-Ro, Seo-Gu(1Ga, Ami-Dong)	Busan 602-739 Korea
0102	IRB / Hallym University Medical Center	2nd bilding, 896, Pyeongchon-dong, Dongan-gu, Anyang	Gyeonggi-do 431-070 Korea
0103	IRB / Konkuk University Hospital	4-12, Hwayang-dong, Gwangjin-gu	Seoul 143-914 Korea

Center No.	Ethics Committee or Institutional Review Board	Department / Organization	Address Country
0200	Local EC Komisja Bioetyczna przy Okręgowej Izbie Lekarskiej w Białymstoku	ul. Świętojańska 7	Białystok 15-082 Poland
0201	Coordinating EC - Komisja Bioetyczna przy Okręgowej Izbie Lekarskiej w Białymstoku	ul. Świętojańska 7	Białystok 15-082 Poland
0203	Local EC - Komisja Bioetyczna przy Okręgowej Izbie Lekarskiej w Lublinie	ul. Chmielna 4	Lublin 20-079 Poland
0301	Ethic Committee of State institution "Institute of Rheumatology RAMS"	34 a Kashirskoe shosse	Moscow 115522 Russia
0302	Ethic Committee of Moscow City Hospital #20	15, Lenskaya str.	Moscow 129327 Russia
0303	Ethic Committee of Moscow 1st City Clinical Hospital	8, Leninsky pr.	Moscow 119049 Russia
0304	Ethic Committee of Medical Academy of Postgraduate Studies	41, Kyrochnaya str.	Saint-Petersburg 193015 Russia
0305	Ethic Committee of City Hospital #25	30, Bolshaya Podyacheskaya str.	Saint-Petersburg 190068 Russia
0306	Ethic Committee City Mariinsky Hospital	56, Litejny pr	St. Petersburg 191104 Russia
0307	Ethic committee of Municipal Institution of HealthCare "Yaroslavl Clinical City Hospital of Emergency call service named after N.V.Soloviev	Zagorodny Sad, 11	Yaroslavl 150003 Russia
0308	Ethic committee of Tver State Medical Academy	4, Sovetskaya str.	Tver 170036 Russia
0309	Ethic committee of Regional Clinical Hospital	1a, Yablochkov str.	Tula 300053 Russia
0400	Etická komisia NÚRCH	Nábrežie Ivana Krasku 4	Piešťany 921 01 Slovakia
0401	Etická komisia FNsP F.D.Roosevelta	Nám. L. Svobodu 1	Banská Bystrica 975 17 Slovakia

Center No.	Ethics Committee or Institutional Review Board	Department / Organization	Address Country
0402	Etická komisia Košica	Košica-Šaca Lúčna 57	Košice 040 15<br>Slovakia
0501	Quorum Review, Inc	1601 Fifth Ave. Suite 1000	Seattle WA 98101<br>Unites States
0502	Quorum Review, Inc	1601 Fifth Ave. Suite 1000	Seattle WA 98101<br>Unites States
0503	Quorum Review, Inc	1601 Fifth Ave. Suite 1000	Seattle WA 98101<br>Unites States
0506	Quorum Review, Inc	1601 Fifth Ave. Suite 1000	Seattle WA 98101<br>Unites States
0507	Quorum Review, Inc	1601 Fifth Ave. Suite 1000	Seattle WA 98101<br>Unites States
0509	Quorum Review, Inc	1601 Fifth Ave. Suite 1000	Seattle WA 98101<br>Unites States
0510	Quorum Review, Inc	1601 Fifth Ave. Suite 1000	Seattle WA 98101<br>Unites States
0512	Quorum Review, Inc	1601 Fifth Ave. Suite 1000	Seattle WA 98101<br>Unites States
0514	Quorum Review, Inc	1601 Fifth Ave. Suite 1000	Seattle WA 98101<br>Unites States
0517	Quorum Review, Inc	1601 Fifth Ave. Suite 1000	Seattle WA 98101<br>Unites States
0517	Quorum Review, Inc	1601 Fifth Ave. Suite 1000	Seattle WA 98101<br>Unites States
0519	Quorum Review, Inc	1601 Fifth Ave. Suite 1000	Seattle WA 98101<br>Unites States
0520	Quorum Review, Inc	1601 Fifth Ave. Suite 1000	Seattle WA 98101<br>Unites States
0600	Taichung Veterans General Hospital Institutional Review Board	40705 No. 160, TaichungKang Rd. Sec. 3	Taichung 40705<br>Taiwan
0601	China Medical University Hospital Institutional Review Board	40447 No.2, Yude Rd., North District	Taichung City<br>40447<br>Taiwan
0602	Chang-Shang Medical University Hospital Institutional Review Board	40201 No.110, Sec, 1, Chien-Kuo N. Road	Taichung 40201<br>Taiwan
0604	Kaohsiung Veterans General Hospital Institutional Review Board	81346 No. 386, Ta-Chun 2st Road	Kaohusing 81346<br>Taiwan
